# Correction: A supramolecular nanovehicle toward systematic, targeted cancer and tumor therapy

**DOI:** 10.1039/d5sc90174j

**Published:** 2025-08-06

**Authors:** Ruizheng Liang, Shusen You, Lina Ma, Chunyang Li, Rui Tian, Min Wei, Dan Yan, Meizhen Yin, Wantai Yang, David G. Evans, Xue Duan

**Affiliations:** a State Key Laboratory of Chemical Resource Engineering, Beijing Laboratory of Biomedical Materials, Beijing University of Chemical Technology Beijing 100029 P. R. China weimin@mail.buct.edu.cn yinmz@mail.buct.edu.cn +86-10-64425385 +86-10-64412131; b Beijing Shijitan Hospital, Capital Medical University Beijing 100038 P. R. China yd277@126.com

## Abstract

Correction for ‘A supramolecular nanovehicle toward systematic, targeted cancer and tumor therapy’ by Ruizheng Liang *et al.*, *Chem. Sci.*, 2015, **6**, 5511–5518, DOI: https://doi.org/10.1039/C5SC00994D.

It has come to the authors' attention that some errors have been found in Fig. 3. The two fluorescence staining images in Fig. 3C were unexpectedly misused due to carelessness when editing the figure. The corrected Fig. 3 is shown below. This correction does not affect the results and conclusions of the study.

**Fig. 3:**
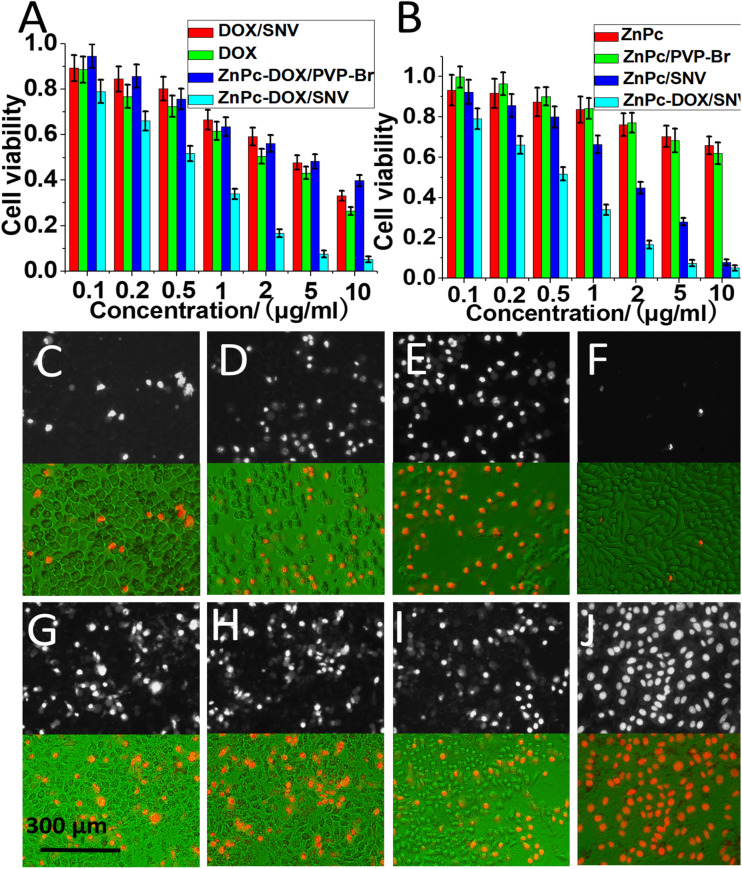
The antitumor performance of (A) DOX/SNV, DOX, ZnPc-DOX/PVP-Br, ZnPc-DOX/SNV, (B) ZnPc, ZnPc/PVP-Br, ZnPc/SNV, ZnPc-DOX/SNV, with a concentration in the range 0–10 μg mL^−1^ after 24 h incubation and 0.5 h irradiation. Fluorescence microscopy and merged images of the HepG2 cells treated with various samples and irradiation (5 μg mL^−1^ and 24 h incubation): (C) ZnPc, (D) ZnPc (5.3%)/PVP-Br, (E) ZnPc (5.3%)/SNV, (F) blank, (G) DOX, (H) DOX/SNV, (I) ZnPc-DOX/PVP-Br, (J) ZnPc-DOX/SNV.

The Royal Society of Chemistry apologises for these errors and any consequent inconvenience to authors and readers.

